# The increasing rate of diabetes in Pakistan: A silent killer

**DOI:** 10.1016/j.amsu.2022.103901

**Published:** 2022-06-03

**Authors:** Saleha Azeem, Ubaid Khan, Ayesha Liaquat

**Affiliations:** a King Edward Medical University, Lahore, Pakistan; b Dow Medical College, Karachi, Pakistan

Diabetes mellitus (DM) is one of the most prevalent public health concerns globally. It is a disorder of carbohydrate metabolism in which blood glucose level is chronically high due to insulin's impaired secretion or action. It has two types: type 1, which occurs in childhood and is usually mediated by immune mechanisms, and type 2, which occurs later in life, particularly with advancing age due to diseases of the pancreas [1]. With the increasing prevalence and mortality associated with it, it is an emotional and economic burden on the patient and a socioeconomic burden on the country's economy. DM is a major cause of cardiac-related deaths, blindness, renal failure, depression, and suicide [2]. Amputations of the diabetic foot have also become increasingly common with the advancing stages of the disease. According to a study carried out in China [3], chronic complications of diabetes were Fig. 1:

**Fig. 1:**
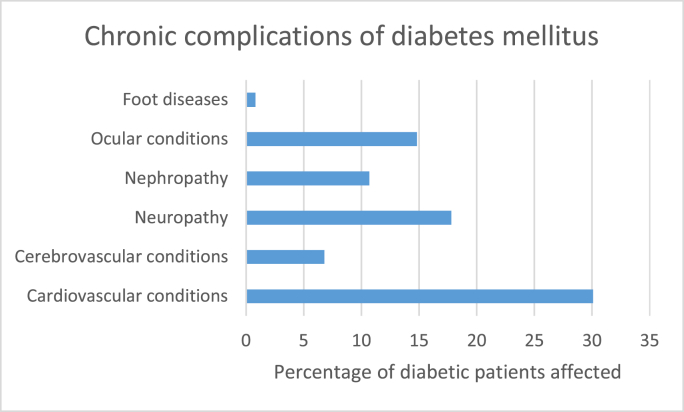
Chronic complications of diabetes mellitus.

Approximately 463 million adults worldwide have diabetes, and 90% of these people suffer from type 2 diabetes mellitus [4]. According to an article by “The News”, Pakistan ranks 3rd in the world in diabetes prevalence after China and India [5]. The prevalence of diabetes in Pakistan in 2016 [6], 2018 [7], and 2019 [4] was 11.77%, 16.98%, and 17.1%, respectively as shown in Fig. 2. According to the International Diabetes Federation, in 2022, 26.7% of adults in Pakistan are affected by diabetes making the total number of cases approximately 33,000,000 [8]. This number is alarmingly high and is also increasing with each passing year. There is also reason to believe that many patients go undiagnosed, making both the actual prevalence and the risk of complications due to the absence of treatment much higher.

**Fig. 2:**
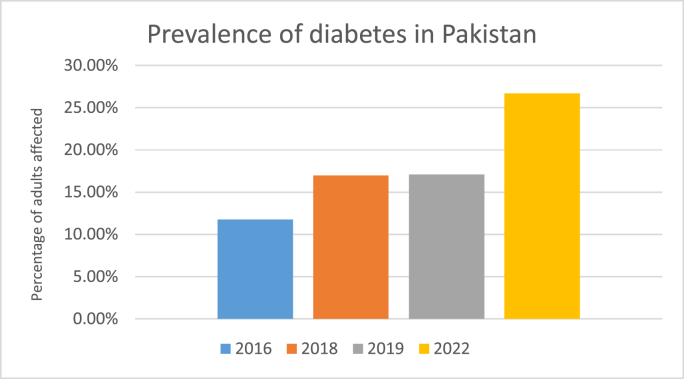
Prevalence of diabetes in Pakistan.

According to the World Health Organization (WHO), diabetes was the largest cause of mortality in 2019, claiming approximately 1.5 million lives [9]. Since the prevalence is greater in low and middle-income countries, Pakistan is one of the more vulnerable countries to diabetes-related deaths.

Factors that predispose individuals, especially adults, to develop diabetes are mainly genetics and lifestyle changes. These include obesity, a sedentary lifestyle, and the intake of more processed food with higher sugar content. Calculated according to the parameters set by WHO Asia-Pacific cutoffs, the overall weighted prevalence of generalized obesity was 57.9% (42% in males and 58% in females), and central obesity was 73.1% (37.3% in males and 62.7% in females) in Pakistan [10]. Similarly, the abundance of canned and highly processed food along with little to no physical activity may even go to the extent of predisposing children to diabetes in the years to come. All of these factors account for the increasing number of pre-diabetics in the country who are always at a risk of becoming diabetic-approximately 10.91% of the adult population as recorded in 2018 [7]. The incidence of diabetes was also found to be significantly more in urban areas (15.1%) as compared to rural areas (1.6%) [11]. The increasing dislocation of people to urban areas from rural areas coupled with the adaptation to the urban sedentary way of life in Pakistan makes the possible increase in the number of cases even more concerning.

National Action Plan for Non-communicable Disease Prevention, Control and Health Promotion in Pakistan (NAP-NCD) is an effort being carried out to prevent and control the incidence of diabetes. This is done by surveillance and maximizing risk factor control. Continued Medical Education (CME) program for healthcare providers and the ensurance of availability of required medicine is also a part of the effort [12]. Numerous management strategies could be employed. Multidisciplinary teams should be created through the capacity building of primary care physicians. Screening methods such as ‘Risk Assessment of Pakistani Individuals for Diabetes’ or ‘RAPID’ should be used for awareness. Further, a nationwide diabetes care program could be implemented that revolves around registration, treatment, and referral protocols [14].

The scarcity of healthcare services in the country, especially in rural areas, is concerning. This may be due to the unfair allocation of funds for the healthcare sector. Most people in Pakistan earn less than $3 per day, which is insufficient to pay for insulin or diabetic medicines [13]. There is a crucial need to increase the number of health care centres and ensure appropriate training of the staff involved. The government must also revise its budget allocation to make treatment more affordable.

Education has always proven to be a powerful tool. While it may not be able to provide treatment to diabetics, it most certainly can spread awareness among the masses regarding its management and prevention. World Diabetes Day, celebrated on November 14, and forums such as Pakistan Diabetes Leadership Forum are examples. Further similar efforts need to be carried out especially involving TV and media, to reach a larger audience. A sedentary way of life needs to be condemned. Instead, adults and especially the elderly should be made aware of the importance of incorporating exercise and diet changes as a part of their daily routine. The government should also keep a check on schools and other outdoor places such as parks, the shrinking of which limits children's physical activity. Such efforts may not be able to bring immediate changes, but they will help us combat the disease over the years to come.

## Ethical approval

This paper did not involve patients, therefore no ethical approval was required.

## Sources of funding

No funding was acquired for this paper.

## Author contributions

**Saleha Azeem:** conception of the study, major drafting of the work, literature search, final approval and agreeing to the accuracy of the work.

**Ubaid Khan:** conception of the study, literature search, major drafting of the work, final approval and agreeing to the accuracy of the work.

**Ayesha Liaquat:** conception of the study, major drafting of the work, final approval and agreeing to the accuracy of the work.

## Consent

This study was not done on patients or volunteers, therefore no written consent was required.

## Guarantor

Saleha Azeem.

## Declaration of competing interest

The authors declare that there is no conflict of interest.
